# Precocious puberty in children

**DOI:** 10.1186/1687-9856-2013-S1-P65

**Published:** 2013-10-03

**Authors:** Irum Atta, Taj Muhammad laghari, Yasir Naqi Khan, Saira Waqar Lone, Mohsina Ibrahim, Jamal Raza

**Affiliations:** 1 National Institute of Child Health, Karachi, Pakistan

## Objective

To determine the etiology of precocious puberty at tertiary care hospital and to compare the clinical and laboratory parameters of central and peripheral precocious puberty.

## Study design

Cross sectional study.

## Place and duration of study

Endocrine clinic at National Institute of child health, Karachi, Pakistan from January 2009 to December 2011.

## Methodology

Children who fulfilled the criteria of precocious puberty were included. Precocious puberty defined as the development of secondary sexual characteristics before the age of 8 years in girls and 9 years in boys. All patients evaluated clinically and on laboratory investigations. Data was entered and analyzed using SPSS version 17.0. Independent sample t-test/ Mann-Whitney U-test were applied.

## Result

Total numbers of patients registered during this period were 84.The conditions causing precocious puberty were central precocious puberty (36.5%), peripheral precocious puberty (38.8%), premature pubarche (10.6%) and premature thelarche (14.1%). In central precocious puberty 26 were female and 5 were male. The causes identified in them were idiopathic (67.74%), hypothalamic hamartoma (12.90%), craniopharagioma (9.67%), arachnoid cyst (3.22%), hypothalamic astrocytoma (3.2%), hydrocephalus (3.2%). In peripheral precocious puberty21 were male and 12 were female. Congenital adrenal hyperplasia (81.8%), adenocarcinoma (9.1%), ovarian teratoma (6.1%) and Mc Cune Albright syndrome (3%) were diagnosed in them. There was difference in the age of onset of puberty of central precocious puberty 3(2-6) versus peripheral precocious puberty 5.25(3.62-7.0). Central precocious puberty children showed higher height SDS, weight SDS, FSH, LH than peripheral precocious puberty.

## Conclusion

Peripheral precocious puberty is more common than central precocious puberty. Height SDS, weight SDS, FSH, LH was higher in central precocious puberty versus peripheral precocious puberty.

